# EMSuite Server: Advanced Tools for Cryo-EM Structure Modeling, Validation, and Refinement

**DOI:** 10.1063/4.0000899

**Published:** 2025-10-27

**Authors:** Joon Hong Park, Javad Baghirov, Xiao Wang, Genki Terashi, Han Zhu, Yuki Kagaya, Pranav D Punuru, Shu Li, Devashish Prasad, Daisuke Kihara

**Affiliations:** 1 Purdue University, West Lafayette, Indiana, USA; 2 University of Maryland, College Park, Maryland, USA; 3 University of Washington, Seattle, Washington, USA

## Abstract

Cryo-electron microscopy (cryo-EM) has advanced structural biology by enabling the determination of numerous macromolecular structures. However, accurate interpretation of cryo-EM maps, particularly at medium to low resolutions, requires sophisticated computational tools to build precise atomic models.

To address this challenge, we present the EMSuite Server (https://em.kiharalab.org, Fig. 1), an integrated web-based platform developed by the Kihara Lab. EMSuite offers a suite of 14 state-of-the-art algorithms for cryo-EM structure modeling, validation, and refinement. These include tools such as DeepMainmast and CryoREAD, which use deep learning for de novo protein and nucleic acid structure modeling. For 5-10 Å resolution maps, the server provides DiffModeler and DMcloud that perform model-map fitting based on diffusion models. For model quality assessment and refinement, the server provides DAQ-Score and DAQ-Refine.

Figure 2 shows examples computed by DeepMainmast and DAQ score using the EMSuite server. As shown in Figure 2A, DeepMainmast accurately built a protein structure model from the cryo-EM map, EMD-1461 (resolution 3.8 Å), achieving an RMSD of 1.30 Å and a TM-score of 0.97 (Fig. 2A). Additionally, DAQ-Score was used to evaluate the local model quality of PDB structure 7fet fitted into EMD-32563. Figure 2B shows color-coded DAQ(AA) scores mapped onto the PDB structure, while Figure 2C presents the plots of the DAQ(AA) scores along the sequence positions. A region around residues 590–620 of chain B indicated negative DAQ scores, suggesting a possible misalignment in the PDB structure model. These visualizations allow users to identify and correct problematic regions in fitted models.

This presentation highlights the EMSuite Server’s key functionalities, including main algorithms such as DeepMainmast, CryoREAD, and the newly released DMcloud. The server integrates deep learning and diffusion-based methods to provide a comprehensive and user-friendly platform for cryo-EM structure modeling, validation, and refinement. We demonstrate how the EMSuite server enables the generation of accurate atomic models from cryo-EM maps. By leveraging these tools, researchers can build high-quality structural models that enhance the understanding of molecular functions and mechanisms.

